# The comforter‐in‐chief: How two traumatic experiences shaped president joe Biden's first 100 days

**DOI:** 10.1111/jopy.12738

**Published:** 2022-06-26

**Authors:** Dan P. McAdams

**Affiliations:** ^1^ Department of Psychology Northwestern University Evanston Illinois USA

**Keywords:** empathy, Joe Biden, narrative identity, psychobiography, redemptive stories

## Abstract

**Objective:**

In the first 100 days of his U.S. presidency, Joe Biden sought to comfort Americans who had lost loved ones to the pandemic and to initiate a surprisingly progressive policy agenda. I interpret these two cardinal features of his early presidency in terms of two traumatic losses in Biden's personal life, contextualizing the argument within a 3‐tiered model of personality.

**Method:**

This psychobiography of a single case mainly follows an inductive, grounded‐theory approach that aims to find patterns in the data that both explain a life and link to evidence‐based constructs in psychological science.

**Results:**

As Biden understands his own life story, the deaths of his wife and daughter in 1972 and first‐born adult son in 2015 forged an empathic sensibility that enables him to connect deeply with other Americans through shared grief and pain. These two traumatic events also inform the uniquely conciliatory approach he followed to instigate social change.

**Conclusions:**

The first 100 days of the Biden presidency provide a striking example of how a particular person's life history comes to meet the broader historical moment. The findings have implications for how personality researchers think about redemptive life stories and the nature of late‐life narrative identity.

## 1 INTRODUCTION

In the November 2020 election, Joseph R. Biden defeated the incumbent Donald J. Trump to become the 46th President of the United States. When he was inaugurated on January 20, 2021, at the age of 78, Biden was the oldest man ever to assume occupancy of the Oval Office. The new President faced a litany of daunting challenges related to the Covid‐19 pandemic, the collapse of the world economy, racial strife in the United States, the mounting ramifications of global climate change, and existential threats to American democratic institutions, the latter starkly exhibited in the January 6 insurrection at the nation's capital. Only hours after the riot at the U. S. Capitol building was quelled, 147 Republicans (139 in the House, 8 in the Senate) voted to overturn Biden's electoral victory, effectively endorsing Donald Trump's false claim that the election was stolen (Yourish et al., 2021). Nonetheless, President Biden promised to bring bipartisanship back to Washington.

In the first 100 days of his administration, Biden worked to reverse many of Donald Trump's initiatives (especially those relating to the environment and climate change), restore confidence and competence in government agencies, oversee the rollout of the Covid‐19 vaccines, diversify the judiciary, ease racial tensions, and improve America's standing in the world community of nations. However, the two signature features that most dramatically marked the early days of his presidency were (1) his heartfelt efforts to comfort a grieving nation devastated by the pandemic (and riven by racial discord) and (2) his dogged pursuit of the most progressive economic policy agenda proposed by a Democratic administration in over 50 years, much of it aimed at helping the poor and bolstering the middle class. As such, Biden embraced two newfound roles. He became (1) a *comforter* for a suffering people and (2) a (reluctant) *revolutionary* for progressive change.

In what follows, I will argue that two searing life events shaped the President's engagement of these powerful roles. The first occurred only weeks before Biden was sworn in as the newly elected United States senator from Delaware, in December of 1972, when his wife and daughter were killed in an automobile accident. The second was the death of his beloved first‐born son, Beau, who at age 46 succumbed to brain cancer. These two events decisively shaped Biden's understanding of himself and the world. They are probably the two most psychologically consequential self‐defining memories in Joe Biden's life (Singer & Salovey, 1993), and they mark dramatic turning points in Biden's internalized and evolving narrative identity (McAdams & McLean, 2013). These tragic events in Biden's life prepared him well to meet an unprecedented moment in American history. The first 100 days of the Biden administration provide a compelling illustration, therefore, of what Erik Erikson (1975) once characterized as the coming together of “life history” and “the historical moment,” or what personality psychologists have more prosaically described as the interaction of the person and the situation (Kenrick & Funder, 1988).

The theoretical framing for this investigation is provided by McAdams and Pals (2006; McAdams, 2013a), who delineate three distinctive layers of personality structure and functioning. Layer 1 refers to the person as a social actor, whose style of enacting social roles is partly determined by broad dispositional personality traits, such as those encompassed within the Big Five framework. Layer 2 refers to the person as a motivated agent, whose goal‐directed strivings are shaped by plans, programs, projects, motives, goals, values, and other contextualized constructs that comprise a person's motivational agenda. Layered over the motivational agenda and the dispositional profile is a person's narrative identity (McAdams & McLean, 2013), which consists of the amalgam of internalized and evolving stories that a person constructs to make sense of their life in time. As the main feature of Layer 3 in personality, narrative identity provides a person's life with some degree of unity, purpose, and a sense of temporal continuity. The current analysis will pay special attention to narrative identity.

As background, I will first provide a capsule summary of Joe Biden's life history. The interpretation itself—both its process and content—will appear under Method and Results. Under Discussion, I will consider implications of my study of Biden for personality research more generally and for the concept of narrative identity.

### 1.1 Early life

Table 1 locates the major events in Joe Biden's personal and professional life on a timeline, running from his birth into a devout Catholic family in 1942 to his inauguration as President of the United States in January of 2021. What the timeline does not quite convey, however, is the emotional feel and tenor of Biden's lived experience. Reports from family members and Biden himself suggest that he was a loved and happy child who was highly esteemed by his peers (Biden, 2008; Witcover, 2010). Tall and athletic, Biden attracted friends and admirers from early childhood onward. “In almost any group, I was the leader,” Biden (2008, p. 22) once wrote, in describing his childhood and teen‐age years.

**TABLE 1:** The life of joe Biden: A timeline

1942	Joseph Robinette Biden, Jr. is born on November 20 in Scranton, Pennsylvania. He is the oldest child in an Irish Catholic family that will eventually include two brothers and a sister
1966	Marries Neilia Hunter; they eventually have three children: Beau, Hunter, and Naomi
1968	Graduates from Syracuse University Law School, ranking 76th in a class of 85 students. Fails one class because he plagiarized a law review article. Admits mistake and chalks it up to not knowing the class rules because he rarely attended class. Begins work with a Wilmington, DE law firm
1972	In a startling upset, Biden defeats a two‐term Republican incumbent to win election to U.S. Senate, running on a moderately liberal platform of promoting civil rights, opposing the Vietnam War, supporting funding for mass transit and health care, and opposing “politics as usual.” Before Biden is sworn in, his wife and one‐year‐old daughter are killed in an automobile accident (December 18), and his two sons are injured
1977	Marries Jill Tracy Jacobs. Daughter (Ashley) born in 1981
1987	Declares candidacy for Democratic presidential nomination but withdraws from the race after being accused of plagiarizing a speech by British Labor Party leader Neil Kinnock. As chairman of the Senate Judiciary Committee, Biden presides over the highly contentious confirmation hearings for Robert Bork, who is President Reagan's nominee for Supreme Court. Biden wins praise for running the hearings with fairness and humor, and for his probing questioning of Bork. He convinces some Republican senators to vote against Bork, who ultimately loses the confirmation vote in the full Senate (42–58)
1988	Suffers a brain aneurism and is hospitalized for months
1991	Presides over Senate confirmation hearings for Clarence Thomas, who is President George Bush's nominee for Supreme Court. A University of Oklahoma law professor, Anita Hill testifies that Thomas sexually harassed her. Biden does not permit testimony, however, from another witness with a similar story, nor from professional experts on sexual harassment, to preserve Thomas's privacy and the hearings' decency
2007	Launches a second bid to gain the Democratic Party nomination for president, but he never breaks beyond single digits in national polls
2009–17	As Vice President, pursues a diversified policy portfolio, with emphasis on foreign policy (especially re the war in Iraq), fiscal policy, and developing positive relations with Congress. Despite criticism from Obama staff regarding Biden's many verbal gaffes, he develops a strong friendship with Obama. In 2012, comes out in favor of same‐sex marriage, before Obama does
2015	Beau Biden dies of brain cancer (May 30)
2020	After finishing fourth in the Iowa caucuses and fifth in the New Hampshire primary, Biden's presidential campaign appears to be on life support. But a key endorsement from an African American South Carolina congressman helps to turn the tide. Biden wins the South Carolina primary by 28 points and then goes on to win 18 of the next 26 contests, securing the Democratic nomination. With record voter turnout during the pandemic, Biden defeats Trump 306–232 in the Electoral College, and wins the popular vote by more than 7 million
2021	Sworn in as 46th president of the United States (January 20)

Biden's father had once been wealthy, but he suffered major financial setbacks around the time Joe was born. Economically strapped, the family moved in with the maternal grandparents. They struggled for many years but eventually secured a relatively stable middle‐ class status when Biden's father became a successful used‐car salesman. Beyond family finances, the only significant adversity that Joe Biden appeared to experience in his early years was his stutter. In the Catholic high school he attended, some of his peers called him “Dash,” because his speech patterns reminded them of Morse code, as in dot‐dot‐dot‐dash. Or, showing off their prowess in Latin, they referred to him as “Joe Impedimenta.” In a scene that stands out in Biden family lore, his mother once marched into the school and threatened to tear off a nun's bonnet after she learned that the nun had made fun of her son's stutter in a literature class. Yet most teachers and other adults were supportive. Biden (2008) wrote:

I beat the stutter with a lot of hard work and with the support of my teachers and my family. But I have never really let go of my impedimenta. It's not a heavy load, but it's always with me, like a touchstone, as a reminder that everybody carries his or her own burdens—most of them a lot bigger than mine—and nobody deserves to be made to feel smaller for having them, and nobody should be consigned to carry them alone. (p. 23)

An indifferent student in college, Biden attended Syracuse Law School, where he continued to receive mediocre grades. He ran into trouble at Syracuse because of his spotty class attendance and his failure to follow instructions on class assignments. In law school, “I was a dangerous combination of arrogant and sloppy,” Biden (2008, p. 36) recalls. Because he failed to properly cite text lifted from an article in the *Fordham Law Review*, Biden was accused of plagiarism on a class paper. A review panel determined that he had not intentionally cheated on the assignment, but he was forced to re‐take the course. The charge of plagiarism would not be the last in his career. During his first run for the presidency (1987–1988), he repeatedly incorporated into his speeches a passage from an address delivered by a British politician, without appropriate attribution. After *New York Times* reporter Marueen Dowd uncovered the truth, Biden attributed the mistake to campaign fatigue. Nonetheless, his opponents saw a pattern, linking the incident to Biden's problems in law school. The conservative columnist William Safire labeled Biden “Plagiarizing Joe.” Because of the allegation and numerous other problems in the campaign, Biden dropped out of the race.

As a law student and young lawyer, Biden relied on his good looks and his winning personal style to get along and get ahead in life. His major interests at the time seemed to be politics and women. Dipping into his $89 tax refund, Biden joined two buddies on a short trip to the Bahamas, where he met, and immediately fell in love with, Neilia Hunter. They married in 1966, and in the next six years produced three children—Beau, Hunter, and Naomi. During this period, local Democratic operatives identified the telegenic and loquacious Biden to be a potential political star, even though he had achieved rather little as a lawyer. With no established politician willing to take the risk, they urged Biden to run for Senate as the Democratic nominee against a well‐respected but less‐than‐dazzling Republican incumbent. Biden's extended Catholic family and many friends campaigned up and down the tiny state of Delaware. In an election year that saw the Republican Richard Nixon overwhelm George McGovern in a landslide, the 29‐year‐old Democrat from Delaware eked out an unexpected victory.

### 1.2 Senator and Vice President

In late November of 1972, Joe Biden was on top of the world. Newly elected to one of the most prestigious jobs on the planet, happily married with three healthy children in tow, a future of seemingly boundless success projecting out to the horizon, Biden was a golden American boy living a Camelot dream, as his political hero John F. Kennedy had lived it a decade before. As with Kennedy, though, tragedy cut the dream short. On her way home from Christmas shopping on December 18, Neilia's car was struck by a tractor‐trailer, instantly killing her and the 13‐month‐old Naomi. For the next five years, Biden held down his Senate job as he endeavored, with some help from his extended family, to be a good father to his two surviving sons. Devastated by the loss of Nelia and Naomi, he believed he would be no more than a one‐term senator. His gloom gradually lifted, however. He began dating Jill Tracy Jacobs, and they were married in 1977. “When I fell for Jill, I started to feel normal again, like I might be capable of running for a second term,” Biden (2008, p. 113) later remarked. Their daughter (Ashley) was born in 1981. Unless he was traveling, Biden rode the Amtrak train home to Delaware nearly every evening, leaving Washington DC in time to spend the night with Jill and the kids.

For the next three decades, Biden served as one of the most influential and publicly visible members of the United States Senate. He specialized in foreign policy and the judiciary. As described in Table 1, presiding over the Supreme Court confirmation hearings of Robert Bork and Clarence Thomas brought him national acclaim and criticism, as did his short‐lived presidential runs for the 1988 and 2008 Democratic Party nominations. In the Thomas confirmation hearings, Biden was criticized for failing to more aggressively pursue charges of sexual misconduct levied against Thomas. He also achieved some degree of notoriety for unfiltered and often ill‐advised public comments—Biden gaffes, as they came to be known. Put simply, he talked too much (even by the standards of American politicians), and he often managed to make offensive comments. Viewed to be an intellectual lightweight early in his career, Biden gradually developed a reputation as a conscientious and energetic legislator who forged positive relationships with senators on both sides of the aisle. He proved to be especially effective in brokering compromises between liberal Democrats and conservative Republicans. Biden famously convinced the arch segregationist Senator Strom Thurmond (South Carolina) to support voting rights legislation in 1982 (Shear et al., 2022). Among his most notable legislative achievements was shepherding through Congress the Violence against Women Act in 1994, for which Biden was able to garner greater than expected support among initially reluctant Republicans.

When he accepted Barack Obama's offer to be his partner on the 2008 Democratic ticket, Biden insisted that, as Vice President, he should be substantively involved in all important aspects of the administration's policy. As Witcover (2010, p. 410) describes it, Biden told Obama: “The good news is, I'm sixty‐five and you're not going to have to worry about my positioning myself to be president. The bad news is, I want to be part of the deal.” Obama agreed to make Biden his chief advisor. The two men developed an effective working partnership over the subsequent 8 years, and a close personal relationship.

Obama gave the eulogy at the funeral for Biden's first‐born son, Beau, who died of brain cancer in the spring of 2015. Biden was devastated by the loss. His energy sapped and spirit nearly broken, Biden refrained from entering the 2016 presidential contest, even though he felt he stood a better chance of defeating a Republican candidate than did Hillary Clinton. He effectively retired from politics at the end of Obama's second term. A year into Trump's presidency, however, Biden began to reconsider. After President Trump refused to condemn white supremacists who marched through Charlottesville, VA in August of 2017, Biden began to lay plans for a third presidential run. In a large field of fiercely liberal Democratic candidates, Biden stood out as a conciliatory moderate who promised to work with Republicans to heal a divided nation and bring positive change for common, hard‐working Americans (Allen & Parnes, 2021). He stumbled badly in the early Democratic primaries, but eventually prevailed to win the nomination, and, in November of 2020, the presidency.

## 2 METHOD

### 2.1 Overall approach

This is a pre‐registered qualitative case study of a single individual. In the pre‐registration process, I spelled out the full plan for the investigation, as described below.

Following the general approach that I developed in previous psychobiographical studies of Presidents George W. Bush (McAdams, 2011) and Donald J. Trump (McAdams, 2017, 2020), my inquiry proceeded in a largely inductive manner. Rather than testing a hypothesis derived from theory or precedent, I began instead with the data on Joe Biden's life and presidency, looking for emerging themes to help me understand the case. When a theme or idea emerged in one data arena (e.g., a magazine article on Biden), I looked for related evidence to support, refute, or elaborate the idea in other data arenas (e.g., Biden's autobiographical writings or speeches). In an inductive, grounded‐theory approach to research, data suggest interpretations, which are then, in turn, evaluated and refined by examining more data (Corbin & Strauss, 2014). The abstract theory/interpretation of the case, then, is discovered within, or emerges out of, the concrete data.

That said, theoretical predilections nearly always guide the process of discovery. As with the previous studies of Bush and Trump, my investigation of Biden's life and presidency was shaped by three decades of research and theorizing within personality and life‐span developmental psychology, wherein I have formulated a three‐tiered model of personality development (McAdams, 2013a; McAdams & Pals, 2006). The theory asserts that personality develops as a patterning of dispositional traits (the person as social actor), personal goals and values (the person as motivated agent), and integrative life stories (the person as autobiographical author), situated within an evolving cultural context. What this meant for the present project was that I was especially sensitive to information that might shed light on Biden's dispositional temperament traits (actor), life goals (agent), and narrative identity (author), as well as the cultural and historical forces that have shaped his life.

### 2.2 Procedure

Once I decided to write a psychobiography of Joe Biden to be considered for publication in this special issue of the *Journal of Personality* (fall of 2020), I followed a four‐step sequence to complete the project, as laid out through the pre‐registration process.

*Step 1: Initial data collection*. In late 2020, I began collecting published information on Joe Biden's life and his public service. This involved finding and reading intellectually reputable and mainly nonpartisan sources (such as news articles and analyses in the *New York Times*, *Wall Street Journal*, and the *Atlantic* and *New Yorker* magazines), published biographies (e.g., Osnos, 2020a; Witcover, 2010), and previous psychological studies of Biden (e.g., Immelman, 2019). Of special interest were Biden's two autobiographical volumes, the first (Biden, 2008) written in preparation for his ill‐fated second run for the presidency, and the second (Biden, 2017) as a meditation on the death of his son. While campaign autobiographies are always self‐serving, I have nonetheless found these documents to be especially revealing of character and narrative identity in past research (see Bush, 1999; Obama, 1995; Trump, 2015). Through June of 2021, I continued to collect and read sources on Biden. I took extensive written notes, compiling an ongoing notebook that included initial observations, comments, and hunches that I developed in reading the sources. The contents of the notebook tracked the inductive and iterative process of gradually formulating a suitable interpretation of Biden's life and presidency.

*Step 2: Articulating a question*. Most psychological studies of the single case ultimately focus on a particular set of questions or issues in a person's life (Elms, 1994; Schultz, 2005). For example, my book‐length study of George W. Bush (McAdams, 2011) focused mainly on the psychological factors that may have influenced his decision, as President, to launch a pre‐emptive invasion of Iraq in 2003. Going into this project, I suspected that whatever the focus would be it would involve paying special attention to the first 100 days of the Biden presidency. As that period unfolded and I collected more and more observations in the notebook, I began to narrow my purview to (1) Biden's response to the pandemic and (2) the unveiling of his surprisingly progressive, even radical, economic policy agenda. I wanted to find psychological antecedents and personal events in his life that might help to explain his characteristic responses to the pandemic and to the economic crisis in America. And I wanted to understand how those processes, events, or determinants from Biden's life history may have been magnified or transformed by the historical moment that Biden confronted in early 2021.

*Step 3: Building an argument*. Even as I was narrowing my focus under the rubric of Step 2 above, I was forming an emotional affiliation for certain features of Biden's life history. Early in my reading, for example, I was struck by Biden's affinity for drawing upon stories that his grandparents and parents told him when he was a child. These seemed to be key to understanding his philosophy of life and certain political positions he adopted. In my notebook, I flagged a powerful account Biden (2008) provided of an incident in 1962 when, as a White lifeguard working at a nearly all‐Black public swimming pool, he came close to engaging in a knife fight with an unruly patron. But Biden, at age 19, managed to defuse the situation. Still, he was ready to do battle; he was not going to back off. I sensed an animating metaphor in all of this, but I could not quite make it work for the purposes of this paper. Nor did the family stories Biden loved to tell bear much fruit for my analysis. Eventually, I decided, instead, to do a deep interpretative dive into the two great tragedies in Joe Biden's personal life—the death of his wife and daughter in 1972, and the death of Beau in 2015. While these events probably influenced Biden in many ways, I thought, they seemed especially germane for interpreting his response to the death and devastation of the Covid‐19 pandemic. Eventually, I came to see these two incidents, and other events that resulted from them, to be instructive for understanding the president's approach to economic policy, as well.

In retrospect, the choice of these two traumatic events may seem too obvious, given the general cultural expectation that psychologists today often focus on the long‐term effects of trauma. For this reason, I initially resisted going down the trauma path. Like me, I suspect that psychobiographers often feel a strong need to come up with something original—an interpretation that goes beyond what others are likely to formulate. That said, sometimes what seems obvious in its importance is indeed important. In the process of writing a psychobiography of Donald J. Trump, for example, I initially downplayed the importance of what nearly every psychologist familiar with Trump's life would underscore: Trump's *narcissism*. However, I eventually came back to the obvious, though I developed a novel conceptual framework for recontextualizing narcissism as a product of Trump's moment‐by‐moment, episodic approach to life (McAdams, 2020).

*Step 4: Composing the manuscript*. To a much greater extent than is the case with hypothesis‐testing, nomothetic research in personality science, writing a psychological biography is itself an integral part of the investigative process. The question (Step 2) and the explanation (Step 3) may be further modified and refined in the process of writing the paper. The limits and affordances of the writing task itself shape not only how the paper is structured but what, in fact, the paper says. In writing certain chapters of my book on Trump (McAdams, 2020), I was unable to predict ahead of time exactly how the argument would unfold. The creative writing process brings new ideas to the fore, which shape the argument in the process of making it.

Let me add that further reshaping occurred as a result the review process and the passing of time between writing the original version of this paper (late summer of 2021) and the revision (January of 2022). In the interim, the editors and two external reviewers provided input that further shaped the paper's argument. By January of 2022, moreover, Biden's presidency passed its one‐year mark. While Biden enjoyed some early success in realizing his policy projects, Congress failed to pass the most progressive features his legislative agenda. As of January 2022, Biden had failed to bring Republicans and Democrats together around common policy goals. And he had failed to elicit the support of all 50 Democratic senators needed to muscle legislation through the budget reconciliation process, or to overturn Senate rules (e.g., the filibuster) that the minority party can use to stymy the will of the majority. These developments cast new light on the limitations of Biden's vision for comity in government—a vision that has its origins in the two traumatic events.

As I see it, psychobiography is *the artful application of psychological science to the understanding of a notable person's life* (McAdams, 2005). It is part science, but also part art. With respect to the art of psychobiography, the author strives to produce something that is aesthetically compelling, even as it incorporates valid scientific theories and findings. This standard does not typically apply to purely scientific articles, of the kind typically published in this journal—though, of course, scientific articles need to be written well enough to be understandable within a scientific community. But the aesthetic of narrative (Bruner, 1986)—the push to tell an engaging story about a specific human being living in a particular time—does (and should) apply, I believe, to the current project, whose character and shape have shifted somewhat as I have engaged in the act of writing.

### 2.3 Positionality

Within qualitative research circles, there is growing interest today in authors' considering and declaring their positionality vis‐à‐vis their subjects. Issues of gender, race/ethnicity, and cohort are especially relevant in this regard. In my case, I am a White, 67‐year‐old male researcher, with over three decades of experience in personality and life‐span developmental psychology and an abiding interest in American culture and politics (e.g., McAdams, 2013b). In this project, I am trying to understand the life and career of a white man who more‐or‐less shares my demographic, though he is 11 years older. In reading Biden's (2008, 2017) autobiographies, I found myself to be intimately and comfortably familiar with many of his references and expressions. Biden reminds me of men I have known, in many ways. More so than for Bush (McAdams, 2011) and Trump (McAdams, 2020), I feel that I identify with Biden. As a result, I may be implicitly relating to him and his life in ways that I have related to important other people in my life, perhaps especially men I have admired.

On the one hand, then, there is bias here, and I am (at least partly) aware of it. I like Biden, and I identify with him. I need to monitor the bias and work hard to rein it in. (A similar, though opposite, problem occurred in my work on Donald Trump, in which I felt a strong antipathy for my subject; I needed to restrain my negative feelings to provide a credible and fair‐minded interpretation of his life.). On the other hand, my positionality vis‐à‐vis Biden may bring to the fore a bias that I can profitably employ as I aim for a deep understanding of my subject. I think I have good insight into his worldview because, in part, I may share it.

## 3 RESULTS

### 3.1 In the aftermath of December 18, 1972

Just 18 days after the death of his young wife and infant daughter, Joe Biden was sworn in as the new senator from Delaware. A black‐and‐white AP photo shows a standing Biden, hand on a family bible, reciting his oath in a Wilmington, DE hospital room, as the four‐year‐old Beau looks on from his bed, his leg elevated in a caste (Lavender, 2015). Biden's face is grim. Even before he settled into his Senate office, Biden threatened to quit the job. It is reported that he contemplated suicide (Osnos, 2020b). Years later, Biden reflected on what he was thinking and feeling in the early months of 1973:

The underpinnings of my life had been kicked out from under me. .. and it wasn't just the loss of Neilia and Naomi. All my life I'd been taught about our benevolent God. This is a forgiving God, a just God, a God who knows people make mistakes. This is a God who is tolerant. This is a God who gave us free will to be able to doubt. This was a loving God, a God of comfort. Well, I did not want to hear anything about a merciful God. No words, no prayer, no sermon gave me ease. I felt God had played a horrible trick on me, and I was angry. I found no comfort in the Church. So I kept walking the dark streets to try to exhaust my rage. (Biden, 2008, p. 81)

In a bipartisan show of comity that is difficult to imagine today, Biden's colleagues—Democratic and Republican—rallied around the new senator, even though many of them hardly knew who he was. Hubert Humphrey called Biden every day, to check in on the young man's state of mind. Senate Majority Leader Mike Mansfield provided fatherly counsel on nearly a daily basis. To distract him from the grief, Ted Kennedy regularly dragged Biden to the Senate gym. President Nixon offered personal condolences. But the kindness shown to Biden offered little solace at first. He wrote: “Losing Neilia and Naomi had taken all the joy out of being a United States senator; it had taken all the joy out of life. Even on the best of days I didn't have the heart to imagine a future within or without the Senate” (Biden, 2008, p. 88).

During his first year in the Senate, Biden felt as if his life had been wrenched away from the flow of time. His immediate past was too painful to think about, and he could imagine no viable future—no future in the Senate, and no future outside of it. It is as if he had stepped outside of his own life story. During this time, Biden experienced a radical disruption in the construction of narrative identity, comprising the third layer of his personality (McAdams & Pals, 2006). As autobiographical authors, people find meaning, purpose, and temporal continuity in their lives by constructing coherent narrative identities (McAdams, 2013a). In Biden's case, he was able still to go through the motions of being a United States senator. He went to committee meetings. He responded to his constituents' mail. Biden continued to perform as if he were the protagonist of an ongoing story. But he was no longer able to narrate the story. He was no longer able to apprehend a viable, long‐term narrative to link who he had been in the past with who he hoped to become in the future.

Biden's recovery was slow and halting. Gradually, he began to make peace with God. And as he did so, he began to re‐assume authorship of his life story. The ideological setting for Biden's life story had always been strongly inflected by his optimistic Catholic faith. A benevolent God looked after His faithful people. Joe was one of God's favorites, blessed with natural talent and positive life circumstances. Violently and suddenly, the automobile accident stripped the original setting away. Eventually, however, Biden replaced what had been destroyed with a more tempered and ambivalent backdrop of personal belief and value, yet one that affirmed life anew:

Somewhere in the middle of all that running around [back and forth between Washington and Wilmington, as well as trips around the country] I began to make my peace with God or with myself. Quite frankly, I just got tired of wallowing in grief. I started to think of my rage at God as an unbecoming form of egotism. What was more self‐indulgent than to think God had been busying himself with my particular circumstances? There's a little cartoon I keep on my desk. In the first panel a guy just struck by lightning, charred, is shaking his fist at God: “Why me!?!” In the second panel God answers with a shrug: “Why not you?”

Why not me? Exactly. Why not me?

Bad things happen. Millions of people had it worse than me. Get up and keep moving, I kept telling myself, and be alive. (Biden, 2008, p. 96)

In Biden's new narrative understanding, God is more‐or‐less indifferent to the plight of humankind. The world seems more dangerous now, and more random. Still, Biden does not abandon his Catholic faith. If anything, his bond with the Church is strengthened. To this day, he regularly attends mass and professes a strong affiliation with Catholicism as a world‐wide community. His humanistic and communal understanding of religion dovetails with a view of human nature that was reinforced in his early Senate years: *People are (mainly) good*. And people *want* to be good.

“I believe basically in the goodness of human nature, and it will win out,” Biden once said, in describing the life of a senator who opposed him on nearly every legislative issue (Witcover, 2010, p. 353). Biden came to believe that people are basically good because of the goodness he saw in his fellow senators. Key to his insight was the extraordinary help his colleagues offered him during his prolonged period of grief. Biden came to love the Senate, and to love individual senators. “I've … seen a thousand small kindnesses from one side of the aisle to the other and hundreds of acts of personal and political courage,” Biden (2008, p. xviii) wrote in the preface of an autobiography. Also, he learned that it is much easier to work with your fellow senators if you like them, and they like you. When the young Biden went to Senator Mansfield with complaints about the Republican Senator Jesse Helms, Mansfield cut him off:

“Listen Joe,” he told me. “Everybody who is here has something. The people who elected them saw something good in them.” Then he told me the story of Helms's adopting a nine‐year‐old with cerebral palsy. … “You're job here is to find the good things in your colleagues—the things their state saw—and not focus on the bad.” (Biden, 2008, p. 110)

Biden drew this moral from the story:

We start with an assumption of goodwill toward one another. The same should hold true in the Senate family, Mansfield had reminded me. It's probably the single most important piece of advice I got in my career. (Biden, 2008, p. 111)

### 3.2 My God, my boy. My beautiful boy

In 2008, Barack Obama commissioned his aide, David Axelrod, to gather background information on Joe Biden, to evaluate Biden's suitability as a running mate. Axelrod came back with this message: “There's something really special about this family. … It's not a thing; it's real. … I don't know how that plays into all of this, but it seems to me to be a real asset” (Osnos, 2020a, p. 58). Axelrod recalled a conversation he witnessed between Joe Biden and his adult son, Beau. After promising to stop by later to see his grandchildren, Joe said goodbye to Beau: “I have a recollection that he kissed him,” Axelrod reported. “He said ‘I love you’” (Osnos, 2020a, p. 58). As a term of endearment, Biden often referred to each of his three children—Beau, Hunter, and Ashley—as “honey” (Biden, 2017, p. 117).

Biden is known for his intimate language and overtly physical displays of affection. He is a hugger and a kisser.^1^ Within the nexus of family affection, Beau may have held the dearest position of anybody, including perhaps even Joe's two wives. In the first‐born Beau, Biden saw an improved version of himself: “Beau Biden, at age forty‐five, was Joe Biden 2.0. He had all the best of me, but with the bugs and flaws engineered out” (Biden, 2017, p. 14). As a member of the Delaware Army National Guard, Beau Biden was deployed in Iraq in 2008. He was elected to be attorney general in Delaware, and he served two terms. Before his illness, Beau had his eye on higher political offices, such as Delaware governor. Joe hoped that Beau might someday become President. Where the father had failed, the favorite son might succeed.

It is expected that parents serve as attachment figures for their children, soothing them when they are agitated and supplying them with a secure base for their explorations of the world (Bowlby, 1969). In the case of Joe and Beau, however, a reciprocal arrangement also seemed to prevail once Beau attained adulthood. “Beau had a way of instilling courage and calming me,” wrote Joe Biden (2017, p. 164). “Beau could always chase my fears away. He saved my life, along with Hunter, forty years ago” after Neilia and Naomi died (Biden, 2017, p. 191). Even when Beau's health was failing precipitously, he served as a source of confidence and courage for his father. “It's going to be okay, Daddy,” he would say. “I'm not going away” (Biden, 2017, p. 191). When Beau died, Joe Biden wrote this in his diary: “It happened. My God, my boy. My beautiful boy” (Biden, 2017, p. 189).

Even the stoic Barack Obama was moved to tears upon the death of Beau Biden. To be expected, Joe was overwhelmed with grief. For months, he and Jill had held out hope that Beau would beat the terrible odds. When the end came, Biden felt more beaten down than angry. Friends remarked that Beau's death erased any vestiges of the young Joe Biden's native cockiness (Osnos, 2020a, pp. 112–113): “The whole Beau experience just killed off the arrogant stuff,” a former aide said. “It was almost physical. You could see it in how he stood.” “He emerges as this sort of humbled, purposeful man.”

The evolution in Biden's disposition as a social actor—from a friendly but cocky young man (who loved to listen to himself speak) to a humbler and deeply empathic older man (who can be remarkably understated and self‐effacing)—roughly tracks the normative developmental trend observed in many longitudinal studies of the Big Five trait *agreeableness* (*A*) (Bleidorn & Hopwood, 2019). While nomothetic research suggests that maturation toward higher levels of agreeableness (and its attendant features of compassion, modesty, and empathy) tends to be gradual, and typically not directly linked to any particular life events in midlife and beyond (Bleidorn et al., 2018), the death of Beau may have, nonetheless, provided further psychological impetus for this particular developmental move in Biden's eighth decade of life.

Biden's acute psychological pain found a precise localized embodiment: “I felt like there was a tiny dark hole in the middle of my chest” (Biden, 2017, p. 199). Biden did not rage against God, as he did after the first big tragedy in his life. Despite God's showing once again the indifference of “Why not you?” Biden still found some solace in his religious faith. “When I pray, I find myself not only praying to God, but praying to Neilia and to my mom to intercede with God for me. It's a way of reminding myself that they are still part of me” (Biden, 2017, pp. 199–200). As for Beau, he retained in death a role he had long played in Joe Biden's life story—as an internalized model of secure attachment, providing the father with what Biden himself (Biden, 2017, p. 192) repeatedly refers to as “home base,” or what Bowlby (1969) and attachment researchers regularly describe as a *secure base* from which to engage the world. In a psychologically literal sense, the son (Beau) was (a nurturing) father to the man (Joe). In this regard, a key scene in Joe Biden's late‐life narrative identity is his reconstruction of a particular conversation he had with his dying son. Beau said:

You've got to promise me, Dad, that no matter what happens, you are going to be all right. Give me your word, Dad, that you are going to be all right. Promise me, Dad.

(Biden, 2017, p. 201).

### 3.3 Comforter‐in‐chief

Since the advent of radio, and perhaps before, Americans have expected their presidents to provide consoling words during times of national trauma. With the strange exception of Donald J. Trump, every president has managed to rise to the challenge. During the Great Depression, Franklin Delano Roosevelt broadcast regular fireside chats, in which he expressed both sympathy for the pain Americans were experiencing and optimism for the future. President George W. Bush gave his greatest speech at the National Cathedral, just days after 9/11, in which he offered words of solace and inspiration for the nation. Even Richard Nixon, dour and depressive in private, was especially solicitous when visiting the families of fallen American soldiers, and he offered consoling words to the young Joe Biden upon the loss of his wife and daughter.

Perhaps no president since Abraham Lincoln, however, has spoken more naturally and more convincingly in a register of human grief than has Joe Biden. His hard‐won gift for this kind of rhetoric stems from the two key traumatic scenes in his own life story. In his first national address as president‐elect, Biden invoked the embodied metaphor at the center of his own grieving: “For families and friends left behind, there's a *gaping hole* in your heart that will never be fully healed” (Baker, 2020). In the same address, Biden observed that the nation has “been living with this pandemic for so long, we're at risk of becoming numb to its toll on all of us” (Baker, 2020). Do not ignore the suffering, Biden counseled. Do not try to repress it, because “our collective pain” will lead to “collective purpose, to control the pandemic, to save lives and to heal a nation” (Baker, 2020).

In February of 2021, the nation passed the half million mark in Covid deaths. To mark the staggering milestone, Biden appeared in the Cross Hall of the White House and pulled a card from his jacket pocket that he said was updated each day with a tally of the dead. Then, he looked into the camera and addressed the survivors directly, alluding several times to the loss of Neilia, Naomi, and Beau:

I know all too well. I know what it's like to not be there when it happens. I know what it's like when you are there holding their hands; there's a look in their eye and they slip away. That black hole in the chest—you feel like you are being sucked into it. The survivor's remorse, the anger, the questions of faith in your soul. (Sanger & Stolberg, 2021)

A few days later, Biden traveled to Texas to comfort victims of a deadly storm. In the words of a *New York Times* reporter, Biden “hugged a little girl who was volunteering [at a food bank], then talked to a woman about the death of his eldest son, once again plugging into the pain of others by accessing his own” (Rogers, 2021a). “Not every president comes naturally to the role of comforting the afflicted,” noted the reporter, “but Mr. Biden, who lost his first wife and has buried two children, is the rare politician who seems to draw strength from the experience.” In early March, Biden gave his first prime‐time White House address, in which he reflected on a “collective suffering, a collective sacrifice, a year filled with the loss of life and the loss of living, for all of us” (Rogers, 2021b). At the end of the speech, Biden pivoted toward hope: “Finding light in the darkness is a very American thing to do,” he said. “In fact, it may be the most American thing we do.” It is important to find the light in the darkness, Biden maintains. And it is important to see the darkness for what it is. Recognizing the 100th anniversary of the 1921 massacre of African American citizens in Tulsa, Oklahoma, Biden told his audience:

For much too long, the history of what took place here was told in silence. While darkness can hide much, it erases nothing. … We do ourselves no favors by pretending none of this ever happened. We should know the good, the bad, everything. That's what great nations do. They come to terms with their dark sides. (Rogers & Shear, 2021)

Joe Biden has never been a great orator. It is reported that once during a particularly long‐winded Biden speech, an irritated Barack Obama passed a note to one of his aides saying: “Shoot. Me. Now.” (Osnos, 2020b). During the 2020 presidential campaign and in the first few months of his presidency, Biden did a better job of keeping remarks concise and to the point, but his oratory rarely soared. Still, he forged strong emotional connections with his audiences when speaking to their pain, and to painful events in American history. In a 2015 interview with late‐night television host Stephen Colbert, Biden spoke of his own grief and listened attentively as Colbert shared the story of the death of his father and two brothers in a plane crash. Colbert later told a journalist that the interaction with Biden “was one of the most compact and affecting conversations I think I've ever had” (Osnos, 2020a, p. 113). Biden “expresses the loneliness of grief and makes you feel less alone,” Colbert said. Biden is keenly aware of his social facility in the arena of human pain. In his own life story, he connects this proclivity explicitly to the death of Beau. As he sees it, helping others deal with their own grief helps him deal with his:

At bottom, I also knew from personal experience, the struggle with grief and pain is an individual event, no matter how many friends and supporters a person can count, no matter how strong his or her faith. But I really believed that just being there, offering my physical presence, letting them see me, still standing, might make a difference to them. Might give them some hope. The simple act of offering solace to other people has been the most rewarding service I have performed in my time out of public office. It seems to be much needed these days. And the truth was, feeling like I could provide some small measure of balm for people in pain has helped me heal. (Biden, 2017, pp. 262–263)

### 3.4 The reluctant revolutionary

From his earliest days in the Senate, Biden positioned himself as a moderate liberal who believed in incremental change. The progressive base of the Democratic Party—who rallied to the words of Ted Kennedy and Jesse Jackson in the 1980s, and who today find common cause with Senator Bernie Sanders and Congresswoman Alexandria Ocasio‐Cortez—has never thrilled to Joe Biden. Even though Biden ran on a progressive Democratic platform, he was widely viewed to be more conservative and more cautious than nearly all his Democratic opponents in the presidential primaries. It was no small shock to the political world, therefore, when President Biden's first 100 days featured a bonanza of progressive legislative initiatives and executive orders. A disgruntled Paul Ryan, former Republican Speaker of the House, summed up the reality of the new Biden presidency this way: “In 2020, the country wanted a nice guy who would move to the center and depolarize our politics,” Ryan said. “Instead, we got a nice guy pursuing an agenda more leftist than any president in my lifetime” (Karni, 2021).

Born in 1970, Paul Ryan never witnessed Lyndon Johnson's Great Society project in the 1960s or Roosevelt's New Deal. Indeed, those are the two apt historical precedents for the scope and the content of Biden's early domestic agenda. In the first two weeks of his administration, Biden signed over 40 executive orders, memoranda, and proclamations enacting or initiating major shifts to the left on racial justice, immigration, climate change, and transgender rights (Lerer & Epstein, 2021). He forcefully advocated for a radically expanded social safety net for Americans, a redistribution of wealth, and massive investment in infrastructure, including what progressives describe as “human” infrastructure, entailing early education, childcare, and health care. In the first 100 days, Biden's big legislative achievement was passing the $1.9 trillion American Rescue Plan Act of 2021, packaged as a stimulus bill in response to the economic destruction wrought by Covid‐19. In truth, it was much more than a stimulus bill, providing substantial economic benefits targeted mainly to the middle class and the poor. Columbia University's Center on Poverty and Social Policy estimated that the bill's provisions, including a generous expansion of tax credits for low‐income Americans with children, would reduce poverty in the United States by more than a quarter for adults and cut the child poverty rate by half (Tankersley, 2021). “The child benefit has the markings of a policy revolution,” wrote Jason DeParle (2021), an expert on families and poverty.

With respect to the psychology of Joe Biden, there are three points to make about his surprising emergence, in 2021, as a strong agent for social and economic change in America.

First, the dramatic and unprecedented events of 2020—especially the mounting deaths and economic destruction caused by the pandemic as well as the racial turmoil that followed the murder of George Floyd in Minneapolis—fed into long‐term disturbing trends in American society (in particular, growing income inequality, the hollowing out of the middle class, disruptions caused by climate change) to create a unique historical moment calling out for change—and Biden recognized the moment. As the *New York Times* columnist David Brooks (2021) put it, Biden “may not have sought transformation, but transformation found him.” He was flexible and opportunistic in this regard, and open to promoting policies that he had never explicitly promoted before.

Second, it seems quite possible that Biden's sensitivity to the suffering of others, reinforced by the two traumatic events in his own life, may have partly motivated him to adopt a more progressive stance toward issues of economics and equity. Biden's aides report that he was “moved by the inequities in pain and suffering that the pandemic has inflicted on the poorest Americans” (Shear et al., 2021). “We all grow,” noted Representative James Clyburn of South Carolina, in reference to Biden's evolution as a change agent. “During the campaign, he [Biden] recognized what was happening in this country, this pandemic,” Clyburn said. “It is not like anything we have had in 100 years. If you are going to address Covid‐19’s impact, you have to address the economic disparities that exist in this country” (Shear et al., 2021).

Third, no matter how revolutionary his policy aspirations may seem, Biden still longed desperately for bipartisan consensus in the first 100 days. Even as progressive Democrats sought to enact the most liberal policy agenda since the 1960s, Biden wanted to bring at least a handful of Republicans on board, especially in the Senate. He wanted this even though not a single Republican voted in favor of the American Rescue Act of 2021. Biden relishes compromise. He loves finding the middle ground (Osnos, 2020a). By dint of temperament and ideology, Biden's aspirations for revolutionary change will always appear to be softer and more mitigated than those of, say, Massachusetts Senator Elizabeth Warren, even when the two see eye to eye on policy. Biden will always seem reluctant in the revolutionary role because he wants to win over those who oppose what he is ultimately trying to achieve.

Biden's dogged efforts to enlist some token Republican support for his policy agenda in 2021 seemed like a fool's errand to many observers. But for Biden the quest for bipartisanship runs deep and long. The headwaters may be traced to the first great tragedy in Biden's life. In the 1970s, Biden came to love the Senate as an institution because, in part, of the extraordinary kindness his fellow senators displayed after the death of his wife and daughter (Biden, 2008; Witcover, 2010). As Biden reconstructs his life story today, both Republicans and Democrats treated Biden like family when he arrived a broken man in the Senate chamber. Even though none of those men and women who served in the Senate in 1973 are there today, Biden still feels a familial tie to the Senate. Families will fight, but they will also work together to achieve common goals. Many of the highlights of Biden's political life, as he reconstructs it today, involve working closely with senators who did not share his beliefs and priorities, and yet finding common ground. The Senators who were part of Biden's “family” over his career “cultivated those [warm and positive] relationships not out of political expediency but because they understood how crucial the bonds of personal relations are in a democracy as jangled and diverse as ours” (Biden, 2008, p. 235). The post‐Trump era in American politics may present the ultimate stress test for Biden's faith in the warm social bonds that, he believes, provide the infrastructural supports for a vital democracy.

At the 100‐day mark in Joe Biden's presidency, he expressed optimism that Congress would succeed in passing a massive package of progressive legislation. With a tiny majority in the House, a 50/50 split in the Senate, and fierce Republican opposition to any policy initiatives smacking of socialism, Biden faced long odds. Nonetheless, in November of 2021, a bipartisan deal was finally struck with respect to infrastructure spending. A total of 19 Republican senators and 13 GOP House members joined nearly all the Democrats to pass a $1 trillion commitment to investing in road and bridge repair, public transit, enhanced broadband, and other public works projects.

During the first year of his presidency, however, Biden and the Democratic Party failed to pass the much larger package of initiatives contained in the Build Back Better legislation. These included investments in universal preschool, the continuation of a childcare tax credit, expansion of health care, initiatives to combat climate change and enhance clean energy technologies, and a slew of other progressive programs and projects. Biden's conciliatory approach toward political opponents and his much‐vaunted negotiating skills proved ineffective on two counts. He was unable to attract any Republican support for Build Back Better. And more critically, he failed to unite fully his own Democratic caucus, even after months and months of painstaking negotiations. Two Democratic senators—Joe Manchin from West Virginia and Kyrsten Sinema of Arizona—repeatedly refused to sign on to the bill, even in various stripped‐down forms. At the one‐year mark of Biden's presidency, therefore, Build Back Better was moribund, and progressive Democrats appeared to be demoralized. Yet the President still expressed optimism that discrete chunks of the legislation, broken off from the overall bill, might eventually garner enough support to win passage in 2022, or after (Shear, 2022; Shear et al., 2022). He also admitted that his affection for the Senate, rooted in personal experiences that go back almost half a century, may not serve him well as President. At a news conference held on January 19, 2022, Biden said:

If I made a mistake, I'm used to negotiating to get things done, and I've been, in the past, relatively successful at it in the United States Senate, even as vice. president. But I think that role as president—is a different role. The public does not want me to be the “president‐senator.” They want me to be the president and let the senators be senators. (Shear et al., 2022)

## 4 DISCUSSION

Ascending to the presidency at a point in history when nearly half a million Americans had already died from the coronavirus, Joe Biden brought a message of solace to the American people. In the first 100 days of his presidency, Biden displayed a remarkable affinity for recognizing and addressing human suffering. In this paper, I argue that Biden's role as comforter‐in‐chief finds its psychological grounding in two traumatic scenes in Biden's life story—the death of his first wife and daughter in an automobile accident in 1972, and the death of his beloved first‐born Beau, who lost his battle with cancer in 2015. As Biden tells it today, these two wrenching experiences forged an empathic sensibility that empowers him to connect deeply with other Americans through shared grief and pain. He knows what the hole in a grieving heart feels like. In public utterances and private conversations, President Biden appears to comfort the afflicted by offering understanding and hope, and in the process, he feels they are helping him to heal as well.

Biden's two traumatic scenes are also implicated, I argue, in the way he has pursued the most progressive policy agenda set forth by any American president in over half a century. Beau's death may have played some role in stamping out the last vestiges of cockiness in Biden's dispositional profile while opening him further to issues of inequity in American society. His embrace of an agenda aimed at strengthening the social safety net, assuring a basic income for all families with children, investing in human infrastructure, addressing racial inequities, and taking on climate change reflects his own political evolution, as well as the opportunities Democrats perceived to be in play early in Biden's term. At the same time, Biden casts himself (for better and for worse) as a *reluctant* revolutionary, desperate to find common ground with his intransigent opponents. Biden's dogged belief in compromise and conciliation reflects his deep love for the United States Senate. And that love continues to be sustained, in part, by the immense gratitude he still feels to those senators, both Democrat and Republican, who rallied around him like family after the death of Nelia and Naomi. Nonetheless, by the end of his first year in office, Biden came to the realization that his love for the Senate has not helped him as much as he originally thought it would in promoting his progressive policy agenda.

The two roles I have foregrounded in this paper—the comforter‐in‐chief and the reluctant revolutionary—emerged and evolved as a function of President Joe Biden's developing personality and the various situations he encountered as president. In the terms of Erikson's (1975) formulation, the two roles reflect the dynamic convergence of life history and the historical moment. A different president with a different set of inclinations and experiences might not have proven so skillful in comforting others during the time of Covid, as we see in shocking counterpoint with respect to Biden's predecessor. Yet, had Biden walked into the Oval Office at a different point in history—say back in 1989 when he made his first run for the presidency, or 2009 when he made his second—the nation might not have been so receptive to a message of heartfelt consolation. The unique meeting of the man and the moment is perhaps most visible in Biden's embrace of a transformative policy agenda in the first 100 days of his presidency. A lifelong moderate, Biden suddenly seemed to be the reincarnation of FDR. Had he changed? Yes, in part. But more importantly, the political environment had changed. Upon passage of the $1.9 trillion Covid stimulus bill, Representative Rosa DeLauro, a Connecticut Democrat, remarked “the moment has found us” (DeParle, 2021). DeLauro proposed a child allowance in ten consecutive Congresses, going back to 2001. In 2021, she—and President Joe Biden—saw her aspiration finally fulfilled.

My psychological biography of Joe Biden foregrounds evidence‐based constructs in personality science that find their conceptual home in the three‐tiered model of personality presented by McAdams and Pals (2006; McAdams, 2013a). For the first layer of the social actor, Biden appears to show the normative developmental trajectory for agreeableness, and perhaps conscientiousness, too (Bleidorn & Hopwood, 2019). From young adulthood through his late 70s, Biden seemed to demonstrate increases in characteristics such as humility, compassion, care, and self‐restraint. When he entered office at the age of 78, Americans saw a warmer and more disciplined Joe Biden than they would have seen, say, 20 years before. From the standpoint of the motivated agent (Layer 2 in personality), Biden demonstrated an interesting reversal of the expected attachment pattern (Simpson & Jones, 2019) with his first‐born son, Beau. As a young man, Beau seemed to serve as a working model of attachment security *for his father*. In this regard, Beau's death may have been doubly painful for Joe Biden—the literal loss of one's beloved son, but also the loss of a nurturing parental figure. With respect to other family members, Biden's attachment patterns are difficult to discern, though he professed love and admiration for both of his parents, and love and devotion to his wife Jill, his surviving younger son Hunter, and his daughter Ashley.

The concept of narrative identity (Layer 3 in personality) looms largest in my analysis, as it did in my previous psychobiographical studies of Presidents Bush (McAdams, 2011) and Trump (McAdams, 2020). As an autobiographical author, Joe Biden has constructed a life story that features two traumatic scenes of sudden and untimely loss. In both cases, Biden finds some redemptive meaning in the negative events (McAdams, 2013b). With respect to the chronology of Biden's narrative identity, both traumatic events are followed by a prolonged and dramatic upward arc. After the death of his wife and daughter, Biden embarks upon a highly successful senatorial career. After Beau's death, Biden comes back to become president. With respect to both events, moreover, Biden engages in *autobiographical reasoning* (Habermas & Bluck, 2000) to find further redemptive meaning in the trauma. Biden learned important lessons about life after the tragedy of 1972, he believes, and he developed an enhanced empathic sensibility after Beau's death.

That said, Biden's take on narrative redemption lacks the triumphalist spirit and sense of destiny‐fulfilled that many American life stories show, especially life stories of midlife American adults who demonstrate high levels of psychological well‐being and strong commitments to promoting the well‐being of future generations (McAdams, 2013b; McAdams & Guo, 2015). As documented in canonical American cultural narratives of atonement, upward social mobility, liberation, self‐improvement, and recovery, well‐functioning midlife American adults tend to view their lives as heroic sagas in which they overcome adversity and fulfill a destiny of self‐determined happiness (McAdams, 2013b). Indeed, George W. Bush's redemptive life narrative roughly followed this pattern, as does Obama's (McAdams, 2013b).

But Biden's story is different. There is more sadness in it, and more gravitas. The protagonist continues to exhibit a strong religious sensibility, but he does not trust God to secure a happy ending. Why torment me, God?! With a shrug, God answers: Why not? Going back to John Winthrop and the Massachusetts Bay Puritans, many Americans have embraced a Protestant perspective on suffering and redemption: Good Christians overcome adversity and are ultimately delivered from their suffering to an enhanced status or state. The Catholic sensibility, however, has often taken a different tack. Christ's willingness to endure suffering on behalf of humanity provides the blessing of unconditional love. Human beings suffer as Christ suffered on the cross. Rather than overcoming suffering to attain a blessing in the end, some versions of Catholicism suggest that suffering is itself the road to grace. A devout Catholic from childhood onward, Biden's perspective on faith seems to resonate with the idea that suffering leads to grace, with Christ's crucifixion providing the ultimate model.

Redemptive life stories affirm hope. And Joe Biden is a hopeful man. But he is cautious, too—and somewhat ambivalent. He has witnessed too much pain to take happiness for granted. While redemptive narratives are usually about overcoming adversity, Biden's story is more about acceptance of adversity, and managing adversity as it comes your way. This kind of narrative identity makes for a humbler and more vulnerable protagonist—but one who seems remarkably well‐suited to offer comfort to other suffering souls.

In the context of contemporary research on the redemptive life stories constructed by highly generative American adults in midlife (Dunlop, 2021; McAdams, 2013b; McAdams & Guo, 2015), Biden's narrative identity describes a more tempered, subtle, and emotionally ambivalent form of redemption than has typically been documented. Positive outcomes do result from negative scenes in Biden's story, but the pain of loss never goes away. The hole in Biden's heart will never be filled. Contra Martin Luther King, the arc of history is long, but maybe it does *not* (automatically) bend toward justice. Or at best, one cannot simply assume that light will prevail over darkness in the long run. You must bear witness to the darkness, Biden insists. You can never ignore the suffering. Still, Biden sustains the fervid hope that the suffering will be redeemed, in some sense. “Finding light in the darkness,” Biden insists, “is the most American thing we do.”

It may be no accident that the oldest man ever to be elected president should offer a perspective that is tempered with caution and with a keen recognition of the dangers and constraints of human life. As he tells it, Biden's awareness of life's precariousness and sense of the tragic dimensions of human existence trace back to the deaths of Neilia and Naomi, when he was still a young man. It seems likely, however, that Biden has continued to process the early loss over the subsequent decades and that it was not until his later years, after the death of Beau, that the full meaning of these tragic events was realized within his narrative identity.

In other words, Biden's construction of a narrative identity that is more about accepting and managing adversity, than it is about fully overcoming adversity, may also reflect how certain older and very mature adults—well past midlife—tend to see the arc of life (McAdams et al., n.d.). One implication, therefore, of this psychobiographical investigation is that personality researchers might do well to investigate the dynamics and development of late‐life narrative identity. To what extent do the culturally favored American narratives of redemption in midlife give way to more tempered life narratives emphasizing acceptance and grace amidst the limitations of life? Is Joe Biden's narrative identity representative of a characteristic late‐life form? Maybe yes. But then again, maybe no, for the *second‐oldest* American ever to assume the office of president was Donald J. Trump.

## 6 AUTHOR CONTRIBUTIONS

As the sole author of this manuscript, the author was responsible for all aspects of the process.

## Endnote

^1^
Over the years, several women have taken offense when Biden has touched them in affectionate ways. For example, a former member of the Nevada legislature complained that she felt demeaned and disrespected when Biden kissed the back of her head (Hayes, 2019). Biden has acknowledged crossing the line of propriety, albeit unwittingly. “It's just who I am,” he has said. “I've always tried to make a human connection. … I shake hands, I hug people, I grab men and women by the shoulders and say you can do this. It's the way I've always been. It's the way I've tried to show I care about them and I'm listening” (Hayes, 2019). After fielding several complaints in 2019, Biden vowed to rein in his public displays of affection and to “be more mindful” of people's personal space. In the spring of 2020, a former Biden staffer named Tara Reade alleged that Biden groped her sexually in 1993. Biden has steadfastly denied Reade's allegation (Glueck et al., 2020).

## References

[jopy12738-bib-0001] Allen, J. , & Parnes, A. (2021). Lucky: How joe Biden barely won the presidency. Crown.

[jopy12738-bib-0002] Baker, P. (2020, December 8). Two presidents, two messages, one killer virus. New York Times.

[jopy12738-bib-0003] Biden, J. (2008). Promises to keep: On life and politics. Random House.

[jopy12738-bib-0004] Biden, J. (2017). Promise me, dad: A year of hope, hardship, and purpose. Flatiron Books.

[jopy12738-bib-0005] Bleidorn, W. , & Hopwood, C. J. (2019). Stability and change in personality traits over the lifespan. In D. P. McAdams , R. L. Shiner , & J. L. Tackett (Eds.), Handbook of personality development (pp. 237–252). Guilford Press.

[jopy12738-bib-0006] Bleidorn, W. , Hopwood, C. J. , & Lucas, R. E. (2018). Life events and personality trait change. Journal of Personality, 86, 83–96. 27716921 10.1111/jopy.12286

[jopy12738-bib-0007] Bowlby, J. (1969). Attachment. Basic Books.

[jopy12738-bib-0008] Brooks, D. (2021, March 25). The Biden revolution rolls on. New York Times.

[jopy12738-bib-0009] Bruner, J. S. (1986). Actual minds, possible worlds. Harvard University Press.

[jopy12738-bib-0010] Bush, G. W. (1999). A charge to keep: My journey to the white house. Harper.

[jopy12738-bib-0011] Corbin, J. , & Strauss, A. (2014). Basics of qualitative research: Techniques and procedures for developing grounded theory (4th ed.). Sage.

[jopy12738-bib-0012] DeParle, J. (2021, April 4). Biden effort to combat hunger marks a profound change.” *New York Times*.

[jopy12738-bib-0013] Dunlop, W. L. (2021). Everything you wanted to know about redemptive stories* (*but were afraid to ask). Journal of Research in Personality, 92, 104078.

[jopy12738-bib-0014] Elms, A. C. (1994). Uncovering lives: The uneasy alliance of biography and psychology. Oxford University Press.

[jopy12738-bib-0015] Erikson, E. H. (1975). Life history and the historical moment. Norton.

[jopy12738-bib-0016] Glueck, K. , Lerer, L. , & Ember, S. (2020, May 1). Biden denies Tara Reade's assault allegation: It never happened. New York Times.

[jopy12738-bib-0017] Habermas, T. , & Bluck, S. (2000). Getting a life: The emergence of the life story inadolescence. Psychological Bulletin, 126, 748–769. 10989622 10.1037/0033-2909.126.5.748

[jopy12738-bib-0018] Hayes, C. (2019, April 3). Joe Biden vows to be “more mindful” on same day as three more women make accusations. *USA Today*.

[jopy12738-bib-0019] Immelman, A. (2019). The political personality of former U.S. vice president joe Biden (Working Paper No. 1.1). St. John's University and the College of St. Benedict.

[jopy12738-bib-0020] Karni, A. (2021, May 27). Paul Ryan critiques Trump's grip on Republican Party. New York Times.

[jopy12738-bib-0021] Kenrick, D. T. , & Funder, D. C. (1988). Profiting from controversy: Lessons from the person‐situation debate. American Psychologist, 43, 23–34. 3279875 10.1037//0003-066x.43.1.23

[jopy12738-bib-0022] Lavender, P. (2015, May 30). Moving photos show a young joe Biden swearing into senate by son Beau's bedside after crash. Huffington Post.

[jopy12738-bib-0023] Lerer, L. , & Epstein, R. J. (2021, February 9). How Biden unified a fractious party under one tent. New York Times.

[jopy12738-bib-0024] McAdams, D. P. (2005). What psychobiographers might learn from personality psychology. In W. T. Schultz (Ed.), Handbook of psychobiography (pp. 64–83). Oxford University Press.

[jopy12738-bib-0025] McAdams, D. P. (2011). George W. Bush and the redemptive dream: A psychological portrait. Oxford University Press.

[jopy12738-bib-0026] McAdams, D. P. (2013a). The psychological self as actor, agent, and author. Perspectives on Psychological Science, 8, 272–295. 26172971 10.1177/1745691612464657

[jopy12738-bib-0027] McAdams, D. P. (2013b). The redemptive self: Stories Americans live by (revised and expanded edition). Oxford University Press.

[jopy12738-bib-0028] McAdams, D. P. (2017). The appeal of the primal leader: Human evolution and Donald J. Trump. Evolutionary Studies in Imaginative Culture, 2, 1–20.

[jopy12738-bib-0029] McAdams, D. P. (2020). The strange case of Donald J. Trump: A psychological reckoning. Oxford University Press.

[jopy12738-bib-0030] McAdams, D. P. , & Guo, J. (2015). Narrating the generative life. Psychological Science, 26, 475–483. 25749704 10.1177/0956797614568318

[jopy12738-bib-0031] McAdams, D. P. , Logan, R. L. , & Reischer, H. (n.d.). Beyond the redemptive self: Narratives of acceptance in later life. Journal of Research in Personality.

[jopy12738-bib-0032] McAdams, D. P. , & McLean, K. C. (2013). Narrative identity. Current Directions in Psychological Science, 22, 233–238.

[jopy12738-bib-0033] McAdams, D. P. , & Pals, J. L. (2006). A new big five: Fundamental principles for an integrative science of personality. American Psychologist, 61, 204–217. 16594837 10.1037/0003-066X.61.3.204

[jopy12738-bib-0034] Obama, B. (1995). Dreams from my father. Three Rivers Press.

[jopy12738-bib-0035] Osnos, E. (2020a). Joe Biden: The life, the run, and what matters now. Scribner.

[jopy12738-bib-0036] Osnos, E. (2020b, August 31). Man in the middle. The New Yorker.

[jopy12738-bib-0037] Rogers, K. (2021a, February 26). Biden visits storm‐battered Texas and vows federal help for the long haul. New York Times.

[jopy12738-bib-0038] Rogers, K. (2021b, March 11). Biden tells nation there is hope after a devastating year. New York Times.

[jopy12738-bib-0039] Rogers, K. , & Shear, M. D. (2021, June 1). Biden promises Tulsa massacre survivors their story will be known in full view. New York Times.

[jopy12738-bib-0040] Sanger, D. E. , & Stolberg, S. G. (2021, February 23). Biden marks Covid milestone in emotional White House ceremony. New York Times.

[jopy12738-bib-0041] Schultz, W. T. (2005). Introducing psychobiography. In W. T. Schultz (Ed.), Handbook of psychobiography (pp. 3–18). Oxford University Press.

[jopy12738-bib-0042] Shear, M. D. (2022, January 20). Biden scales back policy plans and blames G.O.P. obstruction. New York Times.

[jopy12738-bib-0043] Shear, M. D. , Hulse, C. , & Martin, J. (2021, March 11). With relief plan, Biden takes on a new role: Crusader for the poor. New York Times.

[jopy12738-bib-0044] Shear, M. D. , Kanno‐Youngs, Z. , & Rogers, K. (2022, January 21). Biden the negotiator confronts the cold reality of Capitol Hill gridlock. New York Times.

[jopy12738-bib-0045] Simpson, J. A. , & Jones, R. E. (2019). Attachment and social development within a life‐history perspective. In D. P. McAdams , R. L. Shiner , & J. L. Tackett (Eds.), Handbook of personality development (pp. 257–275). Guilford Press.

[jopy12738-bib-0046] Singer, J. A. , & Salovey, P. (1993). The remembered self: Emotion and memory in personality. The Free Press.

[jopy12738-bib-0047] Tankersley, J. (2021, March 6). To juice the economy, Biden bets on the poor. New York Times.

[jopy12738-bib-0048] Trump, D. J. (2015). Crippled America: How to make America great again. Simon & Schuster.

[jopy12738-bib-0049] Witcover, J. (2010). Joe Biden: A life of trial and redemption. William Morrow.

[jopy12738-bib-0050] Yourish, K. , Buchanan, L. , & Lu, D. (2021, January 7). The 147 republicans who voted to overturn election results. New York Times.

